# Conductive Polymer Composites from Renewable Resources: An Overview of Preparation, Properties, and Applications

**DOI:** 10.3390/polym11020187

**Published:** 2019-01-22

**Authors:** Yao Huang, Semen Kormakov, Xiaoxiang He, Xiaolong Gao, Xiuting Zheng, Ying Liu, Jingyao Sun, Daming Wu

**Affiliations:** 1 College of Mechanical and Electrical Engineering, Beijing University of Chemical Technology, Beijing 100029, China; huangyao@mail.buct.edu.cn (Y.H.); s_kormakov@bk.ru (S.K.); heshosha@163.com (X.H.); gaoxiaolong@mail.buct.edu.cn (X.G.); zhengxt@mail.buct.edu.cn (X.Z.); 2 State Key Laboratory of Organic-Inorganic Composites, Beijing 100029, China; liuying@mail.buct.edu.cn

**Keywords:** renewable resources, polymer composites, electrical/thermal conductivity, properties and applications

## Abstract

This article reviews recent advances in conductive polymer composites from renewable resources, and introduces a number of potential applications for this material class. In order to overcome disadvantages such as poor mechanical properties of polymers from renewable resources, and give renewable polymer composites better electrical and thermal conductive properties, various filling contents and matrix polymers have been developed over the last decade. These natural or reusable filling contents, polymers, and their composites are expected to greatly reduce the tremendous pressure of industrial development on the natural environment while offering acceptable conductive properties. The unique characteristics, such as electrical/thermal conductivity, mechanical strength, biodegradability and recyclability of renewable conductive polymer composites has enabled them to be implemented in many novel and exciting applications including chemical sensors, light-emitting diode, batteries, fuel cells, heat exchangers, biosensors etc. In this article, the progress of conductive composites from natural or reusable filling contents and polymer matrices, including (1) natural polymers, such as starch and cellulose, (2) conductive filler, and (3) preparation approaches, are described, with an emphasis on potential applications of these bio-based conductive polymer composites. Moreover, several commonly-used and innovative methods for the preparation of conductive polymer composites are also introduced and compared systematically.

## 1. Introduction

Conductive polymer composites have a range of excellent properties, such as high conductivity, high specific strength, high specific modulus, high temperature, corrosion resistance, fatigue resistance and so on [1,2,3,4,5,6,7]. They can be used not only as a structural material to carry loads, but also as functional materials.

With the application of composite materials and the increase of annual production, a large amount of composite material waste has also been generated. In particular, the high modulus and corrosion resistance of carbon fiber composites has led to the difficulty of disposal and utilization of waste materials [8,9]. The environmental pollution caused by carbon fiber composite materials has attracted extensive attention [10]. Therefore, the technology of recovery and utilization of conductive polymer composites has become an international research hotspot [11,12,13,14].

The production of fillers like carbon fiber requires a lot of energy, so it is very expensive. To recycle and reuse the filler, on the one hand can reduce the production of new carbon fiber energy consumption, and on the other hand, the recycled carbon fiber still has good mechanical properties and utilization value, and can be used in components with relatively low requirements.

There are two main sources of polymer composite waste: one is the waste in the process of production and molding, such as prepreg materials, expired materials, scrap materials, unsuitable parts, flash edges, test waste, etc. [8,15,16]; the other is end-life products. Some developed countries, such as Germany, the United Kingdom, the United States, Japan, and so on, have attached great importance to the development of carbon fiber composite recycling techniques. They have set up special research institutions to solve this problem, and have made some industrial attempts [17,18,19,20]. Potential recycling technologies for polymer composite waste with carbon fillers can be mainly classified into mechanical and chemical recycling. Mechanical recycling comprises mixing some waste materials with original materials and then processing them to form a new material. For carbon fiber reinforced composite waste, carbon fibers are recycled as powders or short fibers, which can only be reused as fillers in the production of new composite materials [21]. In chemical recycling, carbon fillers can be recycled using the following technologies: solvolysis at low temperature, pyrolysis, fluidized bed processing, and solvolysis using near- or super- critical fluids. Carbon fillers can be recycled with good mechanical and surface properties, and then be reused as raw materials in this way. Other methods such as gasification and pyrolysis/gasification can also be used in carbon filler recycling [22]. In this article, the progress of conductive composites from natural or reusable filling contents and polymer matrices including (1) natural polymers, such as starch and cellulose, (2) conductive fillers, and (3) preparation approaches, are described with an emphasis on potential applications [23,24,25]. Moreover, several commonly used and innovative methods for the preparation of conductive polymer composites are also introduced and compared systematically.

## 2. Natural Polymers and Conductive Fillers

### 2.1. Polymers

Natural polymer materials occur widely in animals and plants, in the form of e.g., cellulose, starch, chitin, chitosan, collagen, gelatin and silk. With the increasing demand for materials, synthetic polymer materials began to replace natural polymer materials. However, in recent years, oil resources are decreasing, environmental pollution is becoming more and more serious, and natural polymer materials have received increasing attention by more and more countries.

Natural polymers come from animal, plant and microbial resources in nature, which are inexhaustible renewable resources. These materials are easily decomposed into water, carbon dioxide and inorganic molecules by natural microorganisms or enzymes. They are not only environmentally friendly, but are also biodegradable materials.

#### 2.1.1. Cellulose

Cellulose is highly crystalline, including glucose units linked together in long chains; hemicellulose, as a polysaccharide, acts as a cementing matrix between micro-cellulose fibrils, forming the main structural component of the fiber cell [26].

#### 2.1.2. Starch

Starch-based biodegradable materials with good biodegradability and processability have become a research hotspot in the field of materials.

Whole starch plastics comprise starch molecules which become disordered by adding a small amount of plasticizer and other auxiliaries to form a thermoplastic starch resin. This kind of plastic is the most promising in this category, because it can be completely biodegradable.

Whole starch plastics with starch contents of 90–100% have been developed by the Sumitomo Corporation from Japan, the Warner-lambert Company from USA and the Ferruzzi Company from Italy. The product can biodegrade in one year.

**Figure 1:**
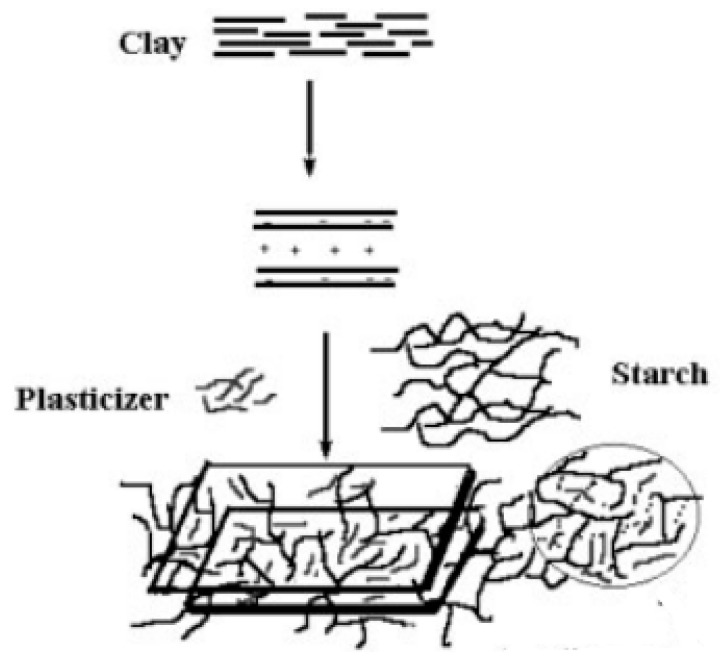
Representation of interactions between plasticizer and starch during migration towards clay galleries (Reproduced with permission [27]).

The sequence of addition of components (starch/plasticizer (glycerol)/clay) has a significant effect on the nature of composites formed, and accordingly, the properties are altered. Glycerol and starch both have the tendency to penetrate into the silicate layers, but the penetration of glycerol is favored, owing to its smaller molecule size. The filler dispersion becomes highly heterogeneous, and the product becomes more brittle when starch is plasticized before filling with clay due to the formation of a bulky structure resulting from electrostatic attractions between starch and the plasticizer. It was concluded that the best mechanical properties can be obtained if a plasticizer is added after the mixing of clay in the starch matrix, as shown in Figure 1 [27].

Recently a new class of hybrid materials of polymers and layered silicates has emerged. Starch has been filled with layered silicates, and an improvement in mechanical and barrier properties was observed [28,29].

#### 2.1.3. Chitin

**Figure 2:**
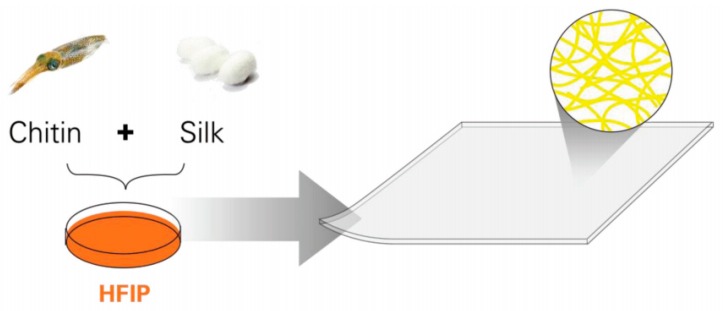
One-step solution based chitin nanofiber silk biocomposite (Reproduced with permission [30]).

Chitin is a rich natural polymer which is widely distributed in low plant fungi, algae cell walls, and arthropods. It has good biocompatibility and biodegradability, and has unique application advantages in the biomedical field. It is of great significance in the construction of a new chitin material for biomedical development. To create the chitin-silk biocomposite, solutions of squid pen β-chitin and B. Mori cocoon silk co-dissolved in hexafluoroisopropanol (HFIP) are dried on a polydimethylsiloxane mold to yield homogeneous films. So, a one-step solution-based chitin nanofiber silk biocomposite that closely replicates the nanostructure of the insect cuticle organic phase, which is made of chitin nanofibers embedded in a silk-like protein matrix, is introduced, as shown in Figure 2 [30].

#### 2.1.4. Protein

Soy protein has been considered recently as an alternative to petroleum polymer in the manufacture of adhesives, plastics, and various binders. Soybean protein, the major component of the soybean, is readily available from renewable resources and agricultural processing by-products. Utilizing these proteins for biodegradable resins will help alleviate environmental problems and add value to agricultural by-products [31,32].

#### 2.1.5. Natural Rubber

The main component of natural rubber is polyisoprene, which comes from latex in the rubber tree. It is a renewable natural resource with excellent comprehensive properties. In order to broaden the application field of natural rubber materials, natural rubber is modified by processes including epoxidation modification, powder modification, resin fiber modification, chlorination, hydrogen chlorination, cyclization and graft modification and blending with other substances.

**Figure 3:**
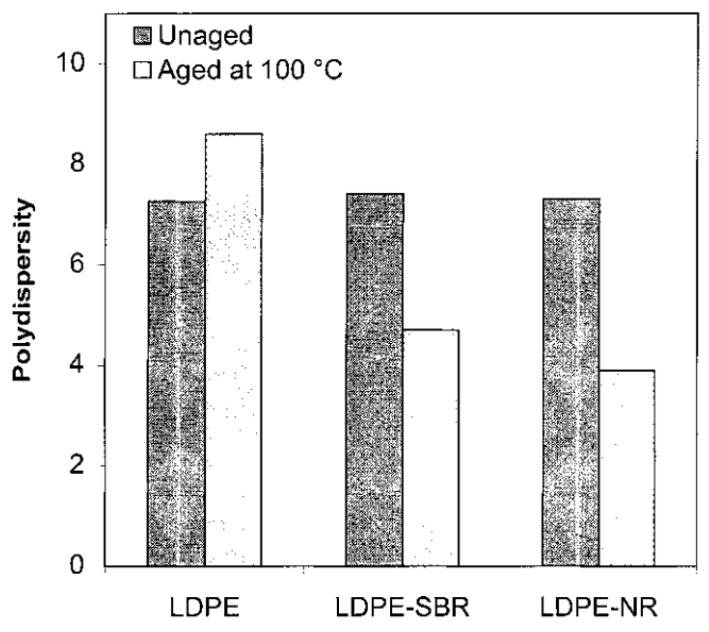
Polydispersity of unaged samples and samples aged at 100 °C for 14 days (Reproduced with permission [33]).

Natural rubber degradable materials were prepared with different preoxidation systems by Albertsson, as shown in Figure 3 [33]. The effect of the modification of silicon on the resilience of natural vulcanized rubber reinforced by silicon and carbon black was studied. The results show that the vulcanized natural rubber containing silane additive recovers elasticity more easily, and the elastic recovery ability increases with the increase of the silane content [34,35,36]. The natural rubber composites of soybean flour were prepared by mixing calcium sulfate as compatibilizer by Wu [37], in which the content of soybean powder and natural rubber was as follows. Natural rubber is uniformly dispersed in a soybean flour matrix, and there are hydrogen bonds between them, which can improve the mechanical properties and water resistance of the material.

#### 2.1.6. Polylactic Acid

In order to reduce pollution and the waste of petroleum resources, the development of environment-friendly, biodegradable materials is a hot research topic at present. Polylactic acid (PLA) is a chemically-synthesized, degradable polymer. Polylactic acid is a kind of polyester plastic. Because of its advantages of good biodegradability and good mechanical properties, it is becoming a hot topic in the research of polymer materials. However, polylactic acid also has its own shortcomings, such as its brittleness, low glass transition temperature, poor impact resistance and high cost, which hinders its commercial application. Therefore, it is necessary to modify polylactic acid physically or chemically.

The composite of conductive fillers such as graphene, carbon black, graphite and biodegradable polylactic acid can make full use of the special properties of conductive fillers such as graphene to improve the performance of polylactic acid.

Graphene nanoparticles/PLA composites were prepared by solution blending of two kinds of graphene nanoparticles (x Gn-25 and N02) with polylactic acid by Mohammad et al. The electrical conductivity of composites is increased by nearly 12 orders of magnitude compared with pure PLA [38].

### 2.2. Conductive Fillers

There are two main kinds of conductive fillers: carbons and metal. Fillers based on carbon include carbon nanotubes (CNT), carbon fibers (CF), and carbon black (CB). For metallic fillers, there are metallic powders, metal flakes, metal-coated fibers and metal nanowire. Table 1 shows the conductivity of metal and carbon fillers [39,40,41].

Different types of fiber waste exist according to the steps taken in the manufacturing process. Fiber waste is produced during the first steps of the production of composite parts. It can also arise from prepreg rolls that did not pass the quality control. After a part is manufactured, processed material waste will be produced that cannot be directly reused, and some parts can be scrapped after quality control. A significant amount of waste is produced during these steps. According to Alex Edge from ELGCF carbon fiber Ltd. in United Kingdom, the majority of the carbon fiber waste that is treated actually arises from these steps. This is due to the long service life of these materials, but also because the waste stream has to be implemented between the dismantling sites and the recyclers.

#### 2.2.1. Carbon Fiber

Carbon fiber as a reinforced fiber of high strength and toughness composite material, with the rapid development of aviation and automobile industry, its demand is also increasing day by day.

In the early stage, the undegradable carbon fiber composite material waste was mainly used in incineration to utilize the thermal energy generated by its combustion. Although this method of recycling was simple and feasible, in the process of incineration, the composite releases a lot of toxic gas, and burying the ash after burning can cause secondary pollution to the soil. As a result, industrial developed countries have strictly prohibited the use of this method to deal with composite waste [8,42].

Carbon materials have long proven themselves as fillers in the manufacture of composite materials based on polymers [43]. The positive qualities of carbon fillers can be attributed to the relative ease of their production and low cost. Moreover, they possess low density, especially in comparison with metals, and have high values of mechanical, electrical and heat-conducting properties, and stability, as well as relative simplicity of storage and easy processing.

Silva’s group [44] prepared green hybrid films by regenerating cellulose/exfoliated graphite nanosheets in ionic liquid. The tensile strength and Young’s modulus of the prepared nanocomposites was improved by 97.5% and 172% respectively after the incorporation of 0.75 and 1 wt % graphite nanoplates. Ruedas and co-workers [45] reported the development of a proprietary formulation based on materials based partially on renewable resources and graphite. The obtained material could be used in the production of capacitive lamps. Wang et al. [46] proposed a novel method of carbon aerogel anode preparation for lithium batteries. Carbon aerogel with large open pores and high surface area can be prepared by the pyrolysis of a three dimensional bacterial nanocellulosic hydrogel construct. Carbon aerogel shows very good electrochemical performance in terms of both the capacity retention and rate performance required for lithium ion batteries.

Lightweight and flexible composite paper has been produced by incorporating nanofibrillated-with-graphite nanoplatelets by Li and co-workers [47]. The authors noted that cellulose, with a high tensile strength but a low thermal conductivity, forms interconnected networks existing in the interspace of graphite nanoplatelets (GNPs), which can significantly improve the tensile strength of the as fabricated composite paper. The hybrid film with 75 wt % GNPs shows an in-plane and through-plane thermal conductivity of 59.46 and 0.64 W/mK, respectively, with a satisfactory tensile strength of 46.39 MPa.

**Figure 4:**
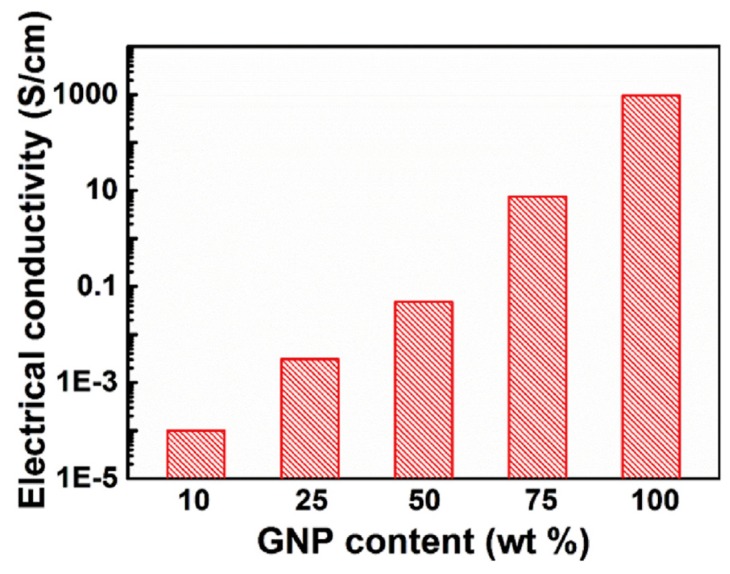
The electrical conductivity of GNP/NFC composite paper measured with different amounts of GNPs (Reproduced with permission [47]).

Figure 4 shows the electrical conductivity of the composite paper as a function of the GNPs amount, which varies by around 7 orders of magnitude, i.e., from 10 wt % GNPs to pristine GNPs papers.

Along with other advantages such as low through-plane thermal conductivity and density, this robust composite paper with superior thermal conductivity promises huge potential applications for heat dissipation. The authors noted that the incorporation of nanofibrillated cellulose (NFC) poses a controllable effect on the electrical and thermal conductivity of the composite paper, while its mechanical strength is dramatically enhanced, revealing the further direction of exploring more advanced thermally-conductive composite paper through tailoring the properties of GNPs, such as by increasing their aspect ratio.

##### High Temperature Pyrolytic Cracking

Pyrolysis is the only commercially-available retracement of carbon fiber reinforced composites, which degrades the composite materials at high temperatures to obtain clean carbon fibers. At the same time, part of the organic liquid fuel can be recovered.

A new technology in which carbon fiber will not be carbonized during heating has been developed by Karborek from Italy, which can obtain carbon fibers of shorter lengths than those of the original material [48].

American adherent technologies had invented a low-temperature and low-pressure thermal decomposition process for carbon fiber composite recycling. The test results show that the surface of the carbon fiber treated by this method is basically undamaged. The strength of the carbon fiber is about 9% lower than that of the original fiber [49,50].

##### Fluidized Bed Process

Fluidized bed thermal decomposition is a carbon fiber recovery method that uses high temperature air heat flux to decompose carbon fiber composites at high temperatures. Usually, this process also uses a cyclone separator to obtain filler particles and clean carbon fibers. The University of Nottingham has carried out a systematic study on the fluidized bed pyrolysis process. The results show that this method is particularly suitable for the recovery and utilization of end-of-life parts of carbon fiber composites containing other mixtures and pollutants [51]. Under the condition that the average particle size of sand in l m/s, fluidized bed is 0.85 mm, the thermal decomposition test of carbon fiber composite is carried out with the fluidized heat flux of 450 °C. The length of carbon fiber recovered was 5.9–9.5 mm. The test results show that the tensile strength of the recycled fiber is about 75% that of the original fiber, but the modulus of elasticity is basically unchanged, so the recovered carbon fiber can partially or completely replace the original short cut carbon fiber.

##### Subcritical and Supercritical Fluids

A team from the University of Validolid in Spain and the University of Nottingham in England studied the chemical recovery of carbon fiber composites using methanol, ethanol and acetone as supercritical fluids. The effects of temperature, pressure, flow rate and alkaline catalyst on the resin decomposition were studied. The results show that the fluid system and alkaline catalyst promote the degradation process and increase the overall reaction rate. By changing the flow rate and the ratio of alkaline catalyst, the resin can be degraded by more than 95% in 15 min, and the recovered fiber can reach a strength of 85–99% of the original [52].

The decomposition process of carbon fiber reinforced epoxy resin composites in supercritical water was studied by Pinero Hemanz R. et al. The results showed that the decomposition rate of epoxy resin was 79.3 by 30 min reaction at 673 K, 28 MPa, and the decomposition rate of epoxy resin reached 95.3 with the addition of a potassium hydroxide (KOH) catalyst. The tensile strength of the obtained carbon fiber can be maintained at 90% that of the original fiber.

#### 2.2.2. Carbon Nanotube

Carbon nanotube (CNT) are linear carbon materials which are grown directly by the catalytic action of low molecular gaseous hydrocarbons, and have higher moduli and strength than ordinary carbon fibers. Conductive properties, such as small size, good flexibility, relatively weak damage in the processing process, and ability to maintain its high aspect ratio, have a wide range of applications in the reinforcement of polymers and the preparation of conductive functional materials.

In order to reduce the percolation threshold of low packing, the better scheme is for the filling to have good dispersion, and not even, but selective distribution, so as to form a two-dimensional conductance network with less content. For the arrangement of CNT in the matrix, Cebeci et al. [53] have studied two kinds of dispersive morphology pairs of CNT in cyclooxygenated lipoids: directed arrangement (A-CNTs) and random distribution (R-CNTs). Their results showed that the conductivity of A-CNTs can reach about 23 s/m, but that of R-CNTs can only reach 10 s/m.

#### 2.2.3. Graphene

Graphene (graphene) is a two-dimensional honeycomb crystal formed by the arrangement of sp2 hybrid carbon atoms. Each carbon atom is connected to the adjacent three carbon atoms by σ bond, and provides an unbonded π electron. This electron can move freely in the plane of graphene, which makes graphene have excellent mechanical and electrical properties. It is widely used in high performance composites, electronic devices and other domains.

In order to reduce the percolation threshold of graphene in polymer, the double percolation structure is used in the compounding of graphene/polymer matrices. Mao et al. [54] found that graphene selectively distributed in the PS phase region when PS/PMMA formed a double continuous phase structure, and the percolation threshold of graphene decreased from 1.5% to 0.5%.

#### 2.2.4. Aluminum Particle

Aluminum and plastic were separated from aluminum-plastic composite film by special processing, and were refined to make plastic particles and aluminum powder. In Brazil, aluminum plastic film is regenerated by plasma technology. This method produces temperatures of up to 15,000 °C by argon electrolysis to obtain liquid aluminum and paraffin. After condensation, aluminum ingots and high-purity stone wax can be formed [55].

#### 2.2.5. Copper Particle

The precipitation potential of copper is much higher than that of other metals. According to the electrochemical theory, it is easier to reduce the electroplating sludge leachate by electrolysis with the potential of more positive metal, so copper ions preferentially reduce at the cathode compared with those of other metals. That is to say, the selective electrochemical separation and extraction of copper in leachate can be realized by electrochemical metallurgy in theory. Researchers taking copper, nickel and other heavy metals as research objects and their sulfate solutions as simulated wastewater, systematically studied the effects of various factors in electrolysis on the current efficiency and recovery rate of metal deposition [56,57,58].

#### 2.2.6. Stainless-Steel Fiber

As a new type of metal fiber, stainless steel fiber (SSF) has been widely studied in the 1980s because of its excellent electrical conductivity and processability. The most outstanding performance of stainless steel fiber is that it is resistant to surface oxidation and rust during high temperature processing, which eliminates the process of complex deoxidization and surface protection. At the same time, it can also maintain conductivity and its electromagnetic shielding function.

The appearance (color), mechanical properties and processing properties of the matrix after adding SSF are the least variable, and a small amount of SSF can achieve reasonable conductivity and electromagnetic shielding efficiency. Polycarbonate (PC), polystyrene (PS) and ethylene-vinyl acetate copolymers (EVA) were filled with stainless steel fibers of about 7 μm in diameter to make conductive composites. When the mass fraction of SSF was 6%, the shielding efficiency could reach 40 dB.

Carbon nanotubes/stainless steel fiber/nylon 6 composites were prepared by a prefabricated master batch method. The material density was less than 1.16 g/mm^3^. The electromagnetic shielding efficiency of the composites increased with the increase of stainless steel fiber content. Percolation occurs in the fiber content from 4 to 6 wt %. When the fiber content reaches 12 wt %, the electromagnetic shielding efficiency is higher than 35db in the range of 30 MHz~1.5 GHz [59].

## 3. Preparation Approaches

Conductive polymer composites are mainly composed of conductive fillers with high conductivity and insulating polymer matrices, in which conductive fillers provide carriers. The carriers transfer into polymer composites by interaction between conductive fillers.

The key to the preparation of conductive polymer composites is how to distribute the filler evenly into the polymer matrix to obtain good processability. The filler can form a conductive network structure in the nanocomposites and provide good conductivity. There are three main methods for preparing conductive polymer composites: melt blending, solution blending and in situ polymerization.

### 3.1. Traditional Compounding Methods

Since the electrical conductivity of the composites was strongly influenced by the volume of conducting filler involved in making conducting paths, the carbon fillers/resin composites were prepared with various volume fractions of conducting fillers and resin. Different dispersion techniques are used to prepare conductive composites (as shown in Table 2). The conductivity of the conductive composite is different due to the filler, matrix and dispersion method.

### 3.2. Organic Molecule Cross-Linking Method

The preparation of crosslinked conductive polymer nanomaterials uses chemical or electrochemical polymerization

The methods of chemical polymerization are usually used as a hard template method (anodic alumina (AAO), polycarbonate (PC), zeolite, thin film as template) and a soft template method (surfactant such as naphthalene sulfonic acid and benzenesulfonic acid, etc.) [74,75,76].

Electrochemically synthesizing cross-linked conductive polymers with nanostructures are prepared by different polymerization methods, such as potentiostatic polymerization, constant-current polymerization, electrodeposition, etc. The reaction is confined to the surface of the electrode and precipitated on the electrode in the form of film. In many cases, nanostructures grow in the direction of an electric field to form a directional structure. The properties and morphology of nanomaterials can be adjusted by electro polymerization conditions, for example, controlling the rate of electrochemical reaction polymerization by controlling the applied voltage or current density, and the amount of the product can also be controlled by the total amount of charge applied in the electrosynthesis. The polymer prepared by the electrochemical method has good morphology, good properties, high conductivity and good stability [77,78].

### 3.3. Spatial Confining Forced Network Assembly (SCFNA)

Constructing a network of conductive fillers in polymeric matrix is essential for the preparation of conductive polymer composites. Although the conductivity of the composites could increase remarkably after the percolation threshold, it is still much lower than expected due to a limited self-assembly interaction between filler particles [79,80,81].

**Figure 5:**
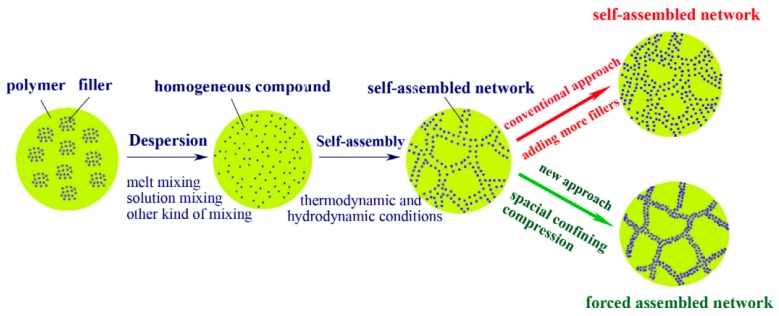
Scheme of technological pathway of SCFNA and conventional compounding method (Reproduced with permission [82]).

High-performance conductive polymer composites could be prepared by the method of spatial confining forced network assembly (SCFNA, Figure 5) [82,83,84]. A homogenous polymer and conductive fillers, prepared by conical twin-screw mixer, was placed in a compression mold with confining space to carry out two-stage compression, free compression and spatial confining compression [85,86]. The electrical conductivity of the SCFNA prepared polypropylene/short carbon fibers was increased to up to 4 orders of magnitude higher than that produced using ordinary compounding technology [87,88].

### 3.4. Intercalation Compounding Methods

Because of the high surface energy, small particle size and high viscosity of the polymer melt, it is not easy to mix evenly. In order to maintain the local order of layered nanometer thermal conductive fillers in the composites, intercalation and recombination technology can improve the thermal conductivity and distribute the polymers among the thermal conductive fillers.

a. Intercalation polymerization

The monomer is dispersed and intercalated into the lamellar layer of the thermal conductive filler, and then the polymerization is initiated and the polymer is formed between the layers, from which the nano-scale recombination is achieved.

b. Polymer intercalation

The polymer solution or melt is mixed with the layered filler, and then the lamellar layer is stripped and dispersed in the polymeric matrix by heating. Polyamide/graphite intercalation composites were prepared by in situ stripping and melting. It was found that the thermal diffusivity of the intercalated composites was much higher than those of polyamide/graphite composites. Higher thermal conductivity may be obtained by using intercalated graphite with a larger scale size and higher expansion ratio [89].

### 3.5. Coating Method

Conductive coating is a cheap, simple process; it can be automatically coated, and is especially suitable for coating the surface of complex shapes, suitable for the purpose of conducting, anti-static and shielding electromagnetic wave. The main research impetus for conductive coatings is to develop conductive coatings with high conductivity, low cost and environmental protection. The waterborne conductive coating has the advantages of low content and low pollution, which greatly save energy and resources; as such, the material has good development prospects.

**Figure 6:**
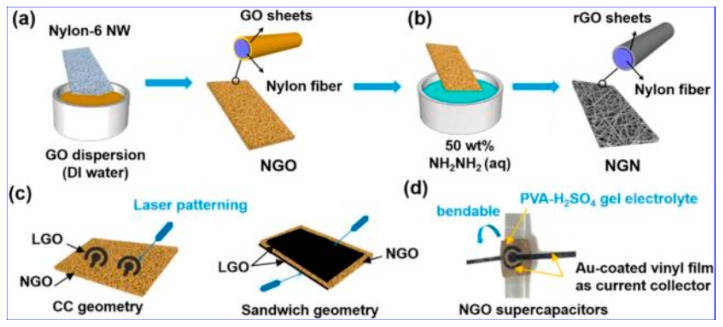
Fabrication process of NGO for conductive and capacitive fabrics (Reproduced with permission [90]).

A conductive nylon-6 nonwoven fabricated via melt-blowing nylon-6 into nonwoven films and dip-coating of the nonwoven matrices with GO dispersions in water is reported by Qin Pan, as shown in Figure 6 [90].

Graphene/cotton composite fabrics were successfully synthesized via a facile “dipping and drying” process followed by a NaBH_4_ reduction method. The flexible 3D conductive network constructed by graphene sheets greatly enhances the conductivity of cotton fabrics [91].

## 4. Multi-Functional Properties of Bio-Based Composites and Their Applications

In recent decades, the demand for biodegradable polymers as environmental friendly packaging materials has increased due to their ability to biodegrade in nature. The term “biodegradable” refers to materials that, under a suitable situation of temperature, moisture, and oxygen availability, are degraded with no adverse environmental impact [92,93]. The importance of renewable products for industrial applications has become extremely clear in recent years, with increasing emphasis on environmental issues such as waste disposal and depleting non-renewable resources. Biopolymers derived from animal (polylactic acid, polyhydroxyalcanoates, polybutylene succinate) or vegetable sources (cellulose-based polymers, alginate, polyisoprene, starch), as well as from bacterial fermentation products (chitin, chitosan, collagen, sericin), have captured the attention of researchers. The materials obtained from the renewable bioresources could be alternatives to petroleum-based synthetic products, due to their advantages of relatively low cost, environmental friendly nature, easy availability, renewability, and nontoxicity. The development of novel materials based on biodegradable polymers is a complex task and is attracting increasing attention in the interests of energy and the environment [94,95,96].

Real applications of biodegradable materials have been limited due to their weak mechanical and thermal characteristics, and filler particles have been indicated to help overcome some of the shortcomings of these films. Biopolymers are generally insulating. To become electrically conductive, polymers should possess a cluster chains of conductive filler, which loosely holds electrons and allows relatively easier delocalization of electrons [77,97].

Currently, there are many composite materials based on the biopolymer matrix that meet the performance requirements. For the manufacture of these materials, a wide range of biopolymer matrices (cellulose, pectin, chitosan, starch, polylactide, etc.) is used. Fillers often use metal particles (copper, silver, aluminum etc.) and their oxides (titanium dioxide, zinc oxide, aluminum oxide etc.) and carbon fillers (carbon black, carbon fiber, carbon nanotubes etc.). Special attention should be paid to materials obtained using nanoparticles. Biopolymers require the incorporation of a nanofiller, and, due to their high production cost and inadequate characteristics, the integration of nanofiller is required to enhance the mechanical strength, electrical conductivity, anti-corrosion, thermal properties etc. [98,99].

These biocomposite materials can be produced in the form of thin films, hydrogels, aerogels, etc., depending on the requirements imposed on them [100,101]. For example, hydrogels prepared from natural polymers have received immense attention due to their safe nature, biocompatibility, hydrophilic properties, and biodegradable nature. Thus, they are considered as good candidates for some potential uses, including as bioconductors, biosensors, bioactuators, electro-stimulated drug delivery systems, as well as neuron-, muscle-, and skin-tissue engineering [102,103,104,105,106]. Aerogels based on piopolymers with the high surface areas and surface charges can be used for the layer-by-layer assembly of conductive polymer composites, carbon nanotubes, titanium dioxide, zinc oxide, and aluminum oxide to enhance the charge capacity, flexibility and mechanical properties for the application in energy storage and other electronic applications [107,108]. Nanocellulose based paper possesses superior optical clarity compared with the regular paper substrate. Transparent and conductive paper can be prepared by the deposition of titanium oxide, carbon nanotubes, silver nanorods, tindoped indium oxide, boron nitride, silica nanoparticles, quantum dot and molybdenum disulfide on nanocellulosic paper substrate [109,110,111,112,113]. Kaolin and a nanocellulose composite could be promising for the cost effective, flexible, low surface roughness and porosity substrate for printed electronic applications [114,115]. Biopolymer-based composites have been used in numerous applications with increasing interest not only due to their renewable, eco-friendly nature, but also because of the flexibility in their processing conditions and competitive cost of their end products [116].

In this section, we will give a more detailed overview of the research aiming at manufacturing and applying conductive biocomposites with different types of fillers.

### 4.1. Carbon Nanotube Based Conductive Biocomposites

One of the most popular nanofillers on the basis of carbon is carbon nanotubes (CNTs). СNTs represent allotropes of carbon with cylindrical structure with a diameter of 1–10 nm, length of several micrometers and high aspect ratio, sometimes reaching values of 10,000 [117,118]. CNTs can be divided into single-walled and multi-walled tubes, depending on the number of their constituent uniaxial carbon cylinders. CNTs were discovered in 1991 [119]. Recent studies have shown that most biopolymer matrices can be successfully used to produce biocomposite materials by incorporating CNTs. The biocompatibility of two main variants of SWCNT and MWCNT has been investigated. One early study revealed that the incorporation of SWCNT into a biodegradable polymeric scaffold did not induce any in vitro cytotoxicity [120]. This type of nanofiller has been successfully electrospun with biopolymers such as chitosan, cellulose triacetate and biodegradable polylactide [121,122]. CNT is used with many types of biopolymers to produce composites with high mechanical and conductive properties [123,124]. It was shown that CNT/biopolymer composites possess excellent mechanical performance. Good biocompatibility and high electrical and electrochemical sensitivity are advantages for implantable biosensor applications. The initial research found that noncovalently functionalzed CNTs could detect serum proteins, including disease markers, autoantibodies, and antibodies [125].

Recently, biocomposites based on CNT and bacterial cellulose have been widely used. Bacterial cellulose (BC), a natural polymer hydrogel, is produced by a primary metabolism process of several genera, such as Agrobacterium, Aerobacter, Salmonella, Escherichia etc. [126]. In recent works, nanofibrillated cellulose has emerged as an alternative for conventionally-used polymers in the fabrication of thermally-conductive papers [127,128]. Among several methods, the integration of a unique carbon nanofiller into the cellulose matrix is regarded as one of the ideal building blocks for the improvement of the mechanical and electrical properties. CNTs have been incorporated into the BC matrix, showing enhanced mechanical strength and electrical conductivity [129,130]. Yoon and co-workers [130] presented the preparation BC/CNT nanocomposite by dipping cellulose pellicles into a multi-walled carbon nanotube (MWCNT) solution. The morphology of the MWCNTs-adsorbed cellulose showed densely adsorbed nanotubes over the surface of the cellulose pellicle. The four-probe electrical measurements of the membrane gave a room-temperature, DC conductivity of approximately 2.0 × 10^−2^ to 1.4 × 10^−1^ S/cm, based on the total cross-sectional area; this result dependent on the amount of MWCNTs in the cellulose membranes.

CNTs, especially SWCNTs possess surface areas which are as large as 2600 m^2^/g, which makes them suitable as drug carriers for biomedical applications. Pantarotto’s group [131] introduced CNTs as a template for presenting bioactive peptides to the immune system. Bcell epitope of the foot-and mouth disease virus (FMDV) was covalently attached to amine group-functionalized CNTs. As a result, the peptides around the CNT adopt the appropriate secondary structure due to the recognition by specific monoclonal and polyclonal antibodies. The immunogenic features of peptide-CNT conjugates were subsequently assessed in vivo. Immunisation of mice with FMDV peptide-nanotube conjugates elicited high antibody responses compared with the free peptide. These antibodies were peptide-specific, since antibodies against CNT were not detected. In addition, the antibodies displayed virus-neutralizing ability. Kim’s group [132] presented a novel, all-solid-state flexible supercapacitor produced by bacterial nanocellulose, carbon nanotubes and ionic liquid based polymer gel electrolytes. The obtained material was characterized by high physical flexibility, desirable electrochemical properties, excellent cyclability and superior mechanical properties.

The incorporation of CNTs as filler materials could be another strategy to enhance the conductive properties of nerve tissue engineered composites. For example, Hasirci’s group [133] demonstrated that MWCNT-poly(2-hydroxyethyl methacrylate) (pHEMA) composite hydrogel conduits could maintain SHSY5Y neuroblastoma cell viability.

Electrical conductivity and electromagnetic interference shielding efficiency of carbon nanotube/cellulose composite paper were evaluated after putting CNT in a continuously interconnected network on cellulose fibers [134]. The produced material was characterized by a volume resistance of 5.3 × 10^−1^ Ω cm.

Farjana’s group [135] reported a flexible conductive sensor based on a conductive biocomposite based on BC and CNT. The authors reported the strain sensitivity of flexible, electrically conductive, and nanostructured cellulose, which was prepared by the modification of bacterial cellulose with double-walled carbon nanotubes. The conductivity of the samples obtained from bacterial cellulose modified with CNT was in the range from 0.034 to 0.39 S/cm. Further, the use of Ionic liquids, 1-butyl-3-methylimidazolium chloride ([BMIM]Cl, 20%), in the production of electrospun hybrid carbon nanotube nanofibers with styrene-acrylonitrile resin showed a significant increase in the conductivity from 1.08 × 10^−6^ to 5.9 × 10^−6^ S/cm for samples containing 3 wt % carbon nanotubes [136,137]. In order to study the sensitivity of the obtained biomaterial, the strain-induced electromechanical response and resistance versus strain were monitored during the application of tensile force.

Research results showed that a mechanically-strong and highly-conductive composite of nanocellulose and carbon nanotube can be prepared from aqueous dispersion in the form of semi-transparent conductive films, aerogels and anisotropic microscale fibers [138,139]. Excellent colloidal stability of aqueous dispersion provided a simple and cost effective method for self-assembly of advanced hybrid nanocomposites for energy applications 

The interaction between DNA and CNT surface has been intensively studied [140,141]. The DNA molecular chain composes of four bases: adenine, cytosine, guanine, and thymine. It has been experimentally and theoretically approved to be of high affinity contact with CNT sidewalls.

**Figure 7:**
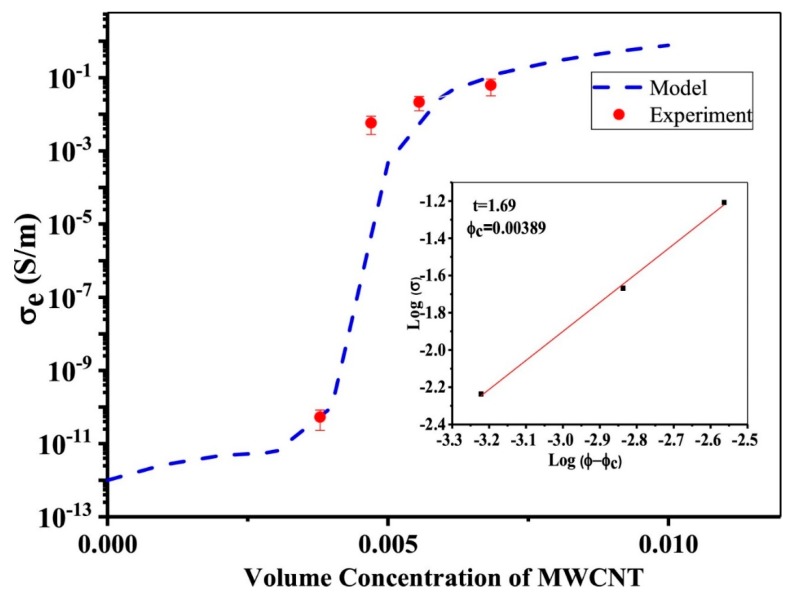
The plot of predicted effective electrical conductivity compared with experimental data for BC/MWCNT nanocomposite aerogels (the inset illustrates the log conductivity against log (ϕ-ϕc)) (Reproduced with permission [142]).

Hosseini et al. [142] applied in-situ biosynthesized bacterial cellulose (BC)/multiwall carbon nanotubes (MWCNTs) nanocomposite hydrogels converted to the conductive nanocomposite aerogels via the supercritical CO_2_ method. The authors obtained a very low percolation threshold value of 0.0041 (volume concentration), predicted for BC/MWCNTs nanocomposite aerogels. The results of measuring the samples’ electro conductivity are presented in Figure 7.

The results of this work indicated that introducing nanotubes BC decreased the average pore size and volume shrinkage of aerogels to 8.6 nm and 2%, respectively. Strain-sensing behavior of the fabricated composite material under tensile loading was also studied. By increasing the strain, the relative resistivity increased and then slightly decreased at critical strain. The strain-sensing behavior over 10 loading/unloading cycles revealed that the max value of ΔR/R_0_ at strain amplitude of 2% was not substantial. On the contrary, a dramatic fall of the max ΔR/R_0_ value observed for strain amplitude of 8% upon cyclic loading unloading. A gauge factor of 21 was obtained for BC/MWCNTs nanocomposite aerogel, indicating the capability of the nanocomposite aerogel as a strain sensor. It is also necessary to note that nanocomposite paper based on nanocellulose with surface carboxylic group sand carbon nanotube has good mechanical properties, flexibility, and excellent electrical conductivity, and is inexpensive and environmental friendly; it therefore has potential for a wide range of flexible electronics applications [143].

Some recent studies have reported on the potential applications of conductive poly(lactic acid) (PLA) compounds for 3D Printing. Lebedev et al. [144] explored the use of carbon nanotubes and natural graphite to increase thermal and electrical conductivity. They achieved increments in the volume resistivity of more than ten orders of magnitude compared with neat PLA. Researchers found that the addition of 0.8% of functionalized carbon nanotubes to the PLA matrix may cause a 78% enhancement in tensile strength and efficient load transfer [145]. The obtained strength is due to the interfacial interaction between the nanotube and polymer matrix which also affects the mobility of polymeric chain.

One of the most interesting directions in the field of Biomedicine is the study of the influence of CNTs functionalized with carboxylic acid, poly-maminobenzene sulfonic acid, and ethylenediamine on the formation of neurites and the growth of neurons [146]. It was noted that the manipulation in surface charge may regulate the neuronal activities. The neurite outgrowth was characterized by the presence of more numerous growth cones, longer neurite length, and enhanced neurite branching when neurons were grown on positively-charged CNTs, as opposed to a neutral or negatively charged filler. In order to facilitate the functionality of CNT- based biomaterials, the incorporation of biological moieties such as growth factor and natural ECM components has also been investigated [147].

Within all those biopolymer functionalized CNTs, Chitosan-wrapped CNT is one of most important for potential applications in variety of fields, such as drug delivery, tissue engineering, electrochemical sensing and actuation. Chitosan also functions as a structural component in the exoskeleton of crustaceans and insects, and is the second most abundant natural biopolymer on earth after cellulose. Owing to its pH-responsiveness and film-forming properties, chitosan can be electrodeposited as a hydrogel on electrodes [148,149]. Chitosan-wrapped CNTs could be directly dispersed at a concentration of 3mg/ml. However, chitosan-wrapped CNT only stabilize in acidic solutions. The solution behavior of chitosan-wrapped CNTs was also investigated [150]. To reveal the influence of electrostatic interaction on the stabilization of chitosan-wrapped CNTs, derivates of chitosan have been used. It was found that the composite material chitosan/CNT has good biocompatibility for the growth of neutral cells [151]. Their suspension coated on a glass carbon substrate could rapidly detect NaDH (t90% < 5s). The sensitivity of chitosan to chemical modifications was used for covalent immobilization of glucose dehydrogenase (GDH) in chitosan/CNT films using glutar dialdehyde (GDI). By dispersing a small fraction of CNTs into a polymer, significant improvements in the mechanical strength of the composite have been also observed. For example, MWCNTs blended with chitosan showed significant improvement in mechanical properties compared with those of chitosan [152]. The composite composed of 2 wt % MWCNT showed a more than doubled Young’s modulus and tensile strength compared to one of neat chitosan. The tuning of the mechanical properties of the polymer can be adjusted depending on CNT loading, and very small amounts may counterbalance the high stability of their structure. Jin et al. [153] noted that incorporating 3% (*w*/*w*) MWCNT into a pHEMA hydrogel did not significantly change cell viability of neuroblastoma, while 6% (*w*/*w*) MWCNT reduced its viability over 7 days of culturing on composite membrane. A relatively high concentration (6% (*w*/*w*)) of MWCNT improved the mechanical property to 0.32 MPa of elastic modulus and conductivity to 8.0 × 10^−2^ Ω/cm, as compared with pHEMA control. The bionanomultilayer biosensor of CNTs and horseradish peroxidase was prepared by a method of layer by layer assembly, and can be successfully applied for the detection of hydrogen peroxide, representing a linear response for hydrogen peroxide from 0.4 to 12 µm with a limit of detection of 0.08 µm [154]. The MWCNTs in the biosensor provided a suitable microenvironment to retain horseradish peroxidase (HRP) activity, and acted as a transducer for improving the electron transport and enhancing the electrochemical signal of the enzymatic reaction product, showing a fast, sensitive and stable response. Paper batteries based on electrodes made with cellulose nanofibrils (CNF)/MWCNTs demonstrated ultrathin electrodes far beyond those accessible with conventional battery technologies. Ultrathin electrodes in combination with readily deformable CNF separators allows for the fabrication of user-friendly paper batteries via origami folding techniques [155]. A composite of MWCNTs-chitosan was used as a material for the entrapment of lactate dehydrogenase onto a glassy carbon electrode in order to fabricate an amperometric biosensor [156]. A CNT-chitosan-lactate dehydrogenase nanobiocomposite film exhibits the abilities to raise current responses, to decrease the electrooxidation potential and to prevent electrode surface fouling. The optimized biosensor for the determination of lactate shows a sensitivity of 0.0083 A M^−1^ cm^−2^ and a response time of about 3 s. The proposed biosensor retained 65% of its original response after 7 days. For the high weight fraction of CNTs in uniform chitosan/CNT in a homogeneous composite electrode chitosan/CNT, the conductivity of the composite electrode could high 34.25 S/cm, which was used to enhance the electrochemical charge-discharge capacity of the bimorphic structure. The bending actuation performance of 15mm long composite strip show 2 mm/s high speed actuation performance under a 3 V low voltage stimulation. These rates are higher than those of most traditional IPMC actuator strips, while no heavy metal element is needed, which is important for biomedical and harptic interface applications.

Farahnaky and co-workers [157] presented a study on the mechanical properties of a biocomposite material based on pectin and CNT. Pectin, a structural heteropolysaccharide, mainly extracted from renewable resources such as citrus fruits and apple pomace, is used in food and non-food systems as a gelling and viscosifying agent. Pectin is a suitable biomaterial for its biodegradability, and has a wide range of applications depending on its degree of esterification and polymerization

**Figure 8:**
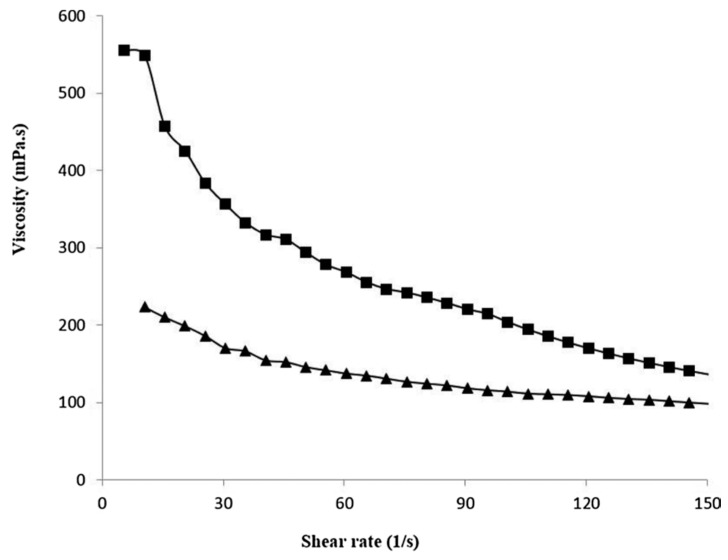
Viscosity-shear rate curves of dispersions (1%) of PM-pectin-CNTs (▲) and CIpectin-CNTs (■) at 25 °C as measured by rotational viscometry (Reproduced with permission [157]).

Viscosity-shear rate curves of dispersions of two types of pectin and carbon nanotubes composites produced by either physical mixing (PM) or chemical interaction (CI) are presented in Figure 8.

The viscosity difference between PM-pectin-CNTs and CI pectin-CNTs was found to be greater at lower shear rates. This is due to greater hydrodynamic volumes of composites of pectin nanocarbon tubes prepared by chemical interaction as compared to physical mixing when dispersed in water.

Biopolymer/CNT composite actuators were initially found to play an important role for smart drug delivery. A novel gelatin-CNTs hybrid hydrogel was synthesized. Cooperation with CNT could maintain the stability of the hybrid hydrogel without crosslinking at 37.8 °C. It has also been noted that the novel hybrid hydrogel, with or without crosslinking, can be used in protein separating. Silk fibroin in the sol state can interact with nanotubes through hydrophobic interactions [158].

To overcome the poor barrier properties and weak mechanical properties of biopolymers, the inclusion of functional fillers with strong physical properties is recommended for the reinforcement of their electrical, mechanical and thermal properties. Special physical and mechanical properties of CNTs make them a suitable candidate for the modification of biodegradable films. This type of nanofiller has been successfully used for different types of biopolymers. This review shows that the addition of CNTs, even in small quantities, leads to a significant increase in the physical and mechanical properties of obtained biocomposites. These materials can find potential applications in many industries and in medicine.

### 4.2. Graphene Based Conductive Biocomposites

Graphene is an example of allotropic 2-dimensional sheet of carbon. Sheets of graphene represent a hexagonal lattice of carbon atoms with thickness of 1 atom. Graphene, like carbon nanotubes, is formed by the organized compounds carbon-carbon, the result of which has a high Young’s modulus and tensile strength. Graphene sheets can be stacked to form three-dimensional graphite, rolled up for the formation of 1D nanotubes and 0D fullerenes [159]. Graphene has been incorporated into polymeric nanocomposites to create advanced materials for flexible electronics, sensors and tissue engineering. Typically, these graphene-based nanofibers are prepared by electrospinning synthetic polymers, whereas electrospun graphene biopolymer nanofibers have been rarely reported due to the poor compatibility of graphene with biopolymers. Graphene has gained particular interest owing to its multifunctional properties such as high specific surface area, electrical and thermal conductivity and superior mechanical strength [160]. Most pertinently, the excellent electrical properties of graphene renders it a promising nanomaterial for novel practical applications such as smart fabrics, nanosensors and flexible electrode materials [161,162,163,164]. The effect of gelatin in collagen reinforced with graphene oxide (GO) was analysed by Bigi’s group [165]. A more than 50% increment in young’s modulus and >60% in fracture stress were observed by adding only 1% of graphene oxide into polymer composite. Also, it should be noted that the addition of graphene oxide can significantly increase the ionic conductivity of the composite material. This filler can be successfully used as a porous separator, which can significantly increase the ion transport. For example, Pan et al. [166] developed an electrospun mat based on GO as a novel solid-state electrolyte matrix, which offers better performance retention upon drying after infiltration with an aqueous electrolyte. Special attention is paid by the authors to the question of how to reduce the ionic conductivity of PVA-based electrolytes upon drying. The developed technology makes it possible to significantly reduce the decay of the ionic conductivity of a material for a month under given conditions, which can be successfully used as a stable power source for flexible electronics.

Sen et al. [167] fabricated 0.1–0.5 wt % graphene nanoplates cellulose composite films using a solution casting method. The 0.25 wt % graphene nanoplates/cellulose composite film produced 0.8 GPa in tensile modulus and 22.6 MPa in strength, an increase of 45% and 31% respectively. The highest electrical conductivity of 5.1 × 10^−3^ S/cm was obtained at 0.50 wt % graphene loading. Peng et al. [168] successfully fabricated graphene-cellulose nanocomposite films by casting, through the exploitation of imidazolium chloride-based Ionic liquids. These cast films showed conductivities of up to 3.2 × 10^−2^ S/cm, thus demonstrating an approach for ionic liquid-biopolymer conductive nanocomposites with graphene.

Javed and co-workers [169] applied cellulose acetate (CA) as biopolymer, graphene oxide (GO) nanoparticles as the source of graphene and 1-butyl-3-methylimidazolium chloride ([BMIM]Cl) as the ionic liquid (IL) to create CA-[BMIM]Cl-GO nanofibers by electrospinning. In this work a new method of exploiting a [BMIM]Cl ionic liquid for the fabrication of graphene-based, bio-inspired (cellulose acetate) conductive CA-[BMIM]Cl-GO nanofibers through electrospinning has been introduced. Combining the advantages of both GO and [BMIM]Cl materials allowed the homogeneous dispersion of GO and better solubility of CA to be achieved. The low concentration of 0.43% graphene oxide into the obtained material enhanced the electrical conductivity of the nanofiber mats by more than four orders of magnitude to 5.30 × 10^−3^ S/cm. The uniform nanostructure of graphite oxide and BMIM in CA nanofibers forms conductive paths, which have been enhanced by chemical reduction of hydrazine via an ultrasonic process.

Wang et al. [128] presented a water-based method to fabricate strong, electrically- and thermally-conductive hybrid thin films from the combination of graphene nanoplatelets and cellulose nanocrystals. Flexible hybrid papers with tunable conductivity as well as good mechanical properties were fabricated. It was noted that the hot-press process can improve the inplane thermal properties up to an optimum addition of cellulose nanocrystal (CNC) loading of 15 wt %, but that this has a negative effect on the through-plane thermal conductivity due to more CNC-GNP contacts being produced. These flexible, electrically- and thermally-conductive hybrid GNP/CNC papers with good mechanical properties may be useful in many applications in the packaging, electrical and heat-conducting fields. Graphene-enhanced laccase-ABTS oxidation showed the pivotal role of graphene for the enzymatic oxidation of the renewable starting material lignin [170]. Graphene served as an effective conductor for electron transfer during the oxidation cycle of laccase-ABTS catalyzed lignin. The outstanding catalytic reinforcement effect makes graphene a candidate for reactivity magnification in enzyme engineering, and can greatly expand the range of applications of carbon-based material. Liang et al. [171] showed a fabrication process of PVA-GO nanocomposite and reported 76% enhancement in tensile strength and 62% in Young’s modulus by the addition of 0.7 wt % of graphene oxide. Nguyen and co-workers [172] noted that composite paper prepared by using nanocellulose and reduced graphene possesses high electrical conductivity, excellent mechanical properties in wet and dry condition, and could have potential applications in humid environments as a conductor, antistatic coating, and electronic packaging. Si and co-workers [173] prepared graphene oxide-bacterial cellulose nanocomposites with a GO content of 0.19, 0.29, and 0.48 wt %. The 0.48 wt % case showed the best mechanical properties with an increase of 38% in tensile strength and 120% in tensile modulus. A relatively low electrical conductivity of 1.24 × 10^−9^ S/cm was noted due to the partial reduction of GO to rGO during the sample preparation. Huang et al. [174] fabricated composite aerogels based on graphene/carboxymethylcellulose for compressive strain sensing evaluation, and a gauge factor (GF) value of 1.58 was obtained.

Feng et al. [175] presented a highly flexible nanocomposite film of bacterial cellulose and graphene oxide with a layered structure produced by the vacuum-assisted self-assembly technique. It was noted that mechanical properties of the BC/GO nanocomposite films depend not only on fibril modulus, but also on orientation and degree of interaction between BC and GO within the film obtained with the assistance of a vacuum. The modulus and tensile strength of the BC/GO film with 5 wt % GO are measured to be 1.7 ± 0.2 GPa and 242 ± 7 MPa, respectively. The values are 10% and 20% higher than those of BC film, indicating that the interaction between BC and GO makes a great contribution to the mechanical enhancement. As discussed above, the oxygen-containing groups on GO can interact with BC through hydrogen bonding. The high aspect ratio of the GO sheets is also favorable for stress transfer. Also, the electrical conductivity of samples was measured. When the RGO content increased from 0.1 to 1 wt %, the conductivity of the film increased by 6 orders of magnitude to 1.1 × 10^−4^ S/m. The conductive properties of the BC-based nanocomposite films make them promising candidates for biosensor and tissue engineering applications.

Composite aerogels were prepared by using carboxymethyl cellulose (CMC) as raw materials, 2D graphene oxide (GO) nanosheets as reinforcement, boric acid (BA) as cross-linker [176]. Composite aerogels with isotropic and anisotropic structures were prepared by controlling the heat transfer rate of the system. The obtained material had a compression strength of 110 kPa at 60% compression, which was 5 times that of the axial and 14 times of the radial of anisotropy structure composite aerogels, and thermal conductivity was also lower than those of the two directions of an anisotropy composite aerogel. The results showed that the mechanical properties of composite aerogels increased with an increase of GO content. When the GO content increased from 0 to 5 wt%, the compressive strength of the composite aerogels increased by 62%, and the Young’s modulus was 3.5 times higher than that of the CMC aerogel. The thermal conductivity of isotropic aerogel (1% BA–5% GO) was as low as 0.0417 W/mK, which was comparable with that of polystyrene foam (0.03–0.04 W/m·K). So, it has the potential to replace traditional insulation materials in thermal insulation.

Nanocellulose and graphene foams having light weight and good combustion efficiency can be used to improve the energy efficiency of buildings [177]. These matrix and conductive electro-active composite materials retain both constituent’s unique responsive properties with nanocellulose as a matrix provide flexibility, while their electroactive properties open possibilities for other applications. The developed composite material has potential applications for the flexible electrodes, flexible display, biocompatible energy scavenging [178]. Zhuo and co-workers [179] presented a carbon aerogel via carbonization of cellulose nanocrystalline/graphene oxide in compression strain and pressure detection. It should be noted that the presence of cellulose plays a critical influence on the viscoelastic properties of the resultant strain sensor. Moreover, the homogeneous dispersion of carbon-based nanofiller within the polymer matrix is vital to improving the performance of sensory materials. Shan et al. [180] fabricated a chitosan-based graphene nanocomposite by a self-assembly approach in an aqueous media. The examined dispersion of GO into chitosan precursor demonstrated an increment of 122% in tensile strength and 64% of Young’s modulus upon the addition of 1 wt % of GO. The T_g_ of nanocomposite increased gradually by the addition of GO component. A glucose biosensor-based chitosan GO nanocomposite was produced, showing good electrocatalytic activity; it also showed good amperometric response at a range of 2–10 mM, and high sensitivity, which makes it a desired candidate for electrochemical detection of glucose [180]. An easily modifiable blend membrane consisting of chitosan and sodium alginate biopolymers forming a poly ion complex with low methanol permeability, high mechanical strength, and high proton conductivity has been used for fuel cell applications due to its abundance in nature and low cost [181,182]. Such membranes are particularly useful in the low to intermediate temperature range. To overcome the low mechanical strength of alginate due to its hydrophilic nature, inorganic fillers or graphene oxide nanocomposites can be used. Hydrogen bonding and high interfacial adhesion between the GO filler and the alginate matrix enhance the thermal and mechanical stabilities. Chitosan-based graphene nanofibers were synthesized by an electrospinning method to improve the electrical conductivity of suspensions [182]. On increasing the loading of GO, a reduction in porosity and permeability of nanofibrous mats occurred, which can be utilized in healing process of wounds. In vivo evaluations showed efficiency in rats. A Brunauer Emmett Teller (BET) test revealed that the incorporation of a GO nanofiller increased the surface are and absorptive properties of the nanocomposite. This green approach showed high absorption efficacy, and can replace petroleum-based membranes. Yang et al. [183] investigated the effect of reduced GO on the electrical conductive properties of PVA nanocomposites, and mentioned the enhancement in conductivity from 6.04 × 10^−3^ to 5.92 S/m on addition of 14 wt % of rGO.

The mechanical and electrical properties of exfoliated graphene and PLA nanocomposite fabricated by melt blending at 175–200 °C temperature were reported by Kim and Jeong [184]. Poly (l-lactic acid) reinforced with GO was fabricated via in-situ polycondensation of lactic acid monomer through lypholization; it was shown that the functionalization of GO improved thermal stability and mechanical properties of the resultant nanocomposite by 105% in tensile strength upon the addition of 0.5 wt % of GO compared to neat PLA, and displayed little effect on crystallinity [185].

### 4.3. Metal Based Conductive Biocomposites

Metallic particles were among the alternative types of fillers for the design of conductivity enhancement of biopolymer-based composite materials. From the fundamental point of view, metallic particles provided strong interest due to their conductivity [186,187,188,189,190]. For example, metallic particles such as Ag, Au and Cu find a wide range of applications. An array of metallic particles can be employed as a pathway for an electrical current. The purpose of the development of technology to generate metal-based polymer biocomposites is to overcome the demerits of polymer matrices in terms of biomedical applications, environmental decontamination, edible packaging applications and many more which has been already reported. The approaches to synthesize metal nanoparticles are chemical vapor deposition (CVD), spray pyrolysis, electrodeposition and chemical methods, sol gel process, rapid solidification, etc. [58,191]. Metal nanoparticles have antibacterial properties, electrical conductivity, optical polarizability and good chemical properties. Metal-based nanocomposites undergo a reduction in size and functionalization of surface, which can also be exploited in plasmonic and sensing applications [192,193].

On the other hand, not only metallic particles but also metal oxide particles still attract attention for their conductivity or polar clustering along structures of composite. Significant efforts have been made with many types of metal oxide such as WO_3_, TiO_2_, ZnO as well as ZrO. From a fundamental point of view, metal oxide particles are categorized as a heterostructure class of material, which refers to the interface that occurs between two layers or region of dissimilar crystalline semiconductor [194,195,196].

Composite biopolymer materials based on titanium dioxide have been widely used in recent years [197,198]. Cellulose nanofibril-TiO_2_ nanoparticle composite membrane electrodes have been reported as membrane electrodes. The novel bio-nanocomposite of chitosan/activated carbon/iron nanoparticles was synthesized via the sonochemical method by Rad’s group [199]. The characterization analysis indicated the successful interactions between oxygen functional groups of activated carbon, amine groups of chitosan and iron ions. The kinetic analysis indicated that the rate-controlling step is a chemical reaction. Adsorption experiments indicate that this bionanocomposite may be an excellent candidate for the removal of heavy metals from industrial wastewaters. Cellulose/polypyrrole and cellulose/polypyrrole-TiO_2_ composites were prepared via in situ oxidative chemical polymerization of pyrrole using FeCl_3_ as an oxidant [200]. Conductive cellulose composites were prepared by the in situ polymerization of pyrrole in the cellulose fiber in the presence of TiO_2_ nanoparticles. TiO_2_ helps to ease the energy crisis through the effective utilization of solar energy based on photovoltaic devices. The authors noted that increasing polypyrrole content in the composite enhanced the dielectric properties of cellulose, where the dielectric properties such as AC conductivity and dielectric permittivity were increased with increasing polypyrrole content in the nanocomposite. The dielectric permittivity (ε’) with frequency revealed that dielectric relaxation at low frequencies was due to the effects of electrode polarization at low frequencies.

Yao et al. [188] reported a flexible Ag/cellulose nanofiber aerogel, obtained from bamboo, with a maximum GF of 1 up to 20% strain. Cellulose films containing different amounts of polyaniline combined with silver nanoparticles were prepared in homogenous conditions by the dissolution of microcrystalline cellulose in 1-butyl-3-methylimidazolium chloride ionic liquid, followed by the mixing with dispersion of silver nanoparticles and polyaniline also prepared in an ionic liquid [201]. The authors obtained a flexible, self-supported film with high electrical conductivity (23–34 S/cm) and homogeneous distribution of constituents. This material can find potential applications as electronic devices, sensors and antimicrobial membranes. Patrycja and co-workers [202] prepared composite films of nanofibrillated cellulose/polypyrrole and nanofibrillated cellulose/polypyrrole-silver nanoparticles for the first time via in situ, one-step chemical polymerization. They found that composites containing silver nanoparticles exhibited electrical conductivity and strong antimicrobial activity. A novel electron conducting biocomposite was synthesized by Perveen et al. [203]. This material was obtained by layer-by-layer assembly of Ppy-Ag-GO/ferritin (Frt)/glucose oxidase (GOx). The presented biocomposite was used to construct a bioanode for glucose-based biofuel cells; it exhibited good electrochemical properties accompanied with considerable stability owing to the synergistic effects between the conductive polymer (i.e., polypyrrole) and silver nanoparticles and graphene oxide. The fabricated bioanode exhibited good electrochemical performance with a maximum current response of 5.7 mA/cm^2^. Zhang et al. [204] prepared solid-state flexible hybrid aerogels based on the combination of cellulose nanofibers (CNF), PANI and AgNP, to be used as an active material for supercapacitors. CNF aerogel was prepared by the freeze-drying of a cellulose nanofibers in aqueous suspension. Next, AgNP were in-situ synthesized in the CNF aerogel in order to make them conductive, and then PANI was electrodeposited onto the Ag/CNF aerogel. The combination of the porous structure of CNF aerogel to the high pseudocapacitive performance of PANI provided an excellent supercapacitor electrode, while the presence of AgNP enabled fast electron transportation channels to achieve high capacitance.

A chitosan-reinforced graphene oxide nanocomposite containing by ZnO may be useful in developing novel antibacterial agents against E. coli and S. aureus bacteria. It can also be utilized as a disinfection agent to inhibit bacterial growth [205]. A high performance nanometric metallic material for Li-ion batteries involving the use of cellulose was made by reducing SbCl_3_ with NaBH4 in the presence of commercial cellulose fibers [206]. Wang and co-workers [207] described the production of a flexible electrode for high performance superconductors, composed of a hybrid film of PANI, AgNP and exfoliated graphite (ExG). The fabrication process involved firstly the electrochemical exfoliation of graphite rods in order to produce the ExG, followed by aniline chemical polymerization in a dispersion containing ExG, cellulose and silver nitrate. The as-prepared mixture was then vacuum-filtered and dried, yielding a self-supported thin film. The ExG/aniline ratio influenced the capacitance, conductivity, rate capability and cyclic stability. Despite this, studies describing the production of self-supported electrical conductive films composed of CEL/PANI/AgNP are still scarce.

## 5. Conclusions

In summary, the preparation, properties, and applications of conductive polymer composites from renewable resources have been reviewed in this paper. An enormous volume of synthetic polymers accumulating in the natural environment has become a major threat to the planet due to their poor degradability. Recent research has highlighted the potential applications of biocomposites using eco-friendly approaches. These types of materials have many interesting properties (such as nontoxicity, biodegradability, renewability, biocompatibility etc.) which make them ideal candidates for many potential applications, including chemical sensors, light-emitting diodes, batteries, fuel cells, heat exchangers, biosensors, and so on.

Biocomposites are gradually becoming a realistic substitute for conductive polymer composites. Because biocomposites are entirely or partially derived from renewable resources, producing them on a large-scale can significantly reduce the cost of materials. Recent advances in obtaining and recycling natural or reusable filling contents and polymers, and new preparation techniques for composites have provided significant opportunities for the use of renewable resources to improve valuable additional materials, and have enhanced support for global sustainability. Further, biocomposites derived from bacterial synthesis have been shown to be promising biomaterials for various biomedical applications such as tissue engineering, drug delivery, wound dressing, cardiovascular applications, etc.

Natural filling contents and polymers are biodegradable, but conductive polymer composites based on renewable resources can be designed to be biodegradable, or not, according to specific application requirements. Their unique properties and potential applications would undoubtedly bring new market opportunities in the 21st century.

## Figures and Tables

**Table 1:** The conductivity of metal and carbon fillers.

Filler Type	Electrical Conductivity (S/cm)	Thermal Conductivity (W/mK)	Density (g/cm^3^)
Aluminium	3.538 × 10^5^	234	2.7
Copper	5.977 × 10^5^	386–400	8.9
Silver	6.305 × 10^5^	417–427	10.53
Nickel	1.43 × 10^5^	88.5	8.9
CNTs	3.8 × 10^5^	2000–6000	2.1
CF	10^2^~10^5^	10–1000	1.5~2.0
Graphene	6000	4000–7000	1.06
Graphite	10^4^	100–500	2.25
Aluminium nitride	˂10^−13^	100–319	3.235
Boron nitride	10^−14^	185–400	2.27

**Table 2:** Different dispersion techniques for the preparation of conductive composites.

Matrix	Filler	Dispersion Technique	Max. Conductivity S/cm @ Filler Concentration	Reference
Epoxy	Graphite	High speed mixer	124 @ 75 vol % graphite	[60]
PPS	Graphite	Melt mix	73 @ 80 wt % graphite	[61]
Epoxy	Graphite	Melt mix	53 @ 80 wt % graphite	[62]
COC	CF	Melt mix twin screw	1.2 × 10^−2^ @ 60 phr CF	[63]
Epoxy	CF	Melt compounding	6.34 @ 80 wt % CF	[64]
epoxy resin	CF	chemical vapor deposition	0.022	[65]
LDPE	Copper	Internal mixer	0.11 @ 24 vol % Copper + 76 vol % LDPE	[66]
HDPE	Silver	Melt mix, twin screw	0.01 @ 24 vol % silver + 76 vol % HDPE	[67]
HDPE	Aluminum	Melt mix	10^−2^ @ 55 vol % Aluminum + 45 vol % HDPE	[68]
HDPE	Copper	Melt mix	10^−5.7^ @ 55 vol % Copper + 45 vol % HDPE	[68]
HDPE	Iron	Roll mill		[69]
PVDF	Zinc	Solution mix	5 × 10^−4^ @ 50 vol % zinc + 50 vol % PVDF	[70]
SBS	Copper nanowires	Vacuum filtrated	1858 @ 20 wt % CUNWS + 80 wt % SBS	[71]
PVC	Copper	Dry mix, hot press	10^3.8^ @ 38 vol % copper + 62 vol % PVC	[72]
PS	Silver	In-situ bulk polymerization	103 @ 20 wt % silver + 80 wt % PS	[73]

## Abbreviations

SCFNA	spatial confining forced network assembly
HFIP	hexafluoroisopropanol
GNP	graphite nanoplatelets
NFC	nanofibrillated cellulose
KOH	potassium hydroxide
PPS	polyphenylene sulfide
SSF	stainless steel fiber
COC	copolymers of cycloolefin
CF	carbon fiber
LDPE	low-density polyethylene
HDPE	high-density polyethylene
PVDF	polyvinylidene fluoride
SBS	styrene-butadiene-styrene block copolymer
PVC	polyvinyl chloride
PS	polystyrene
AAO	anodic alumina
PC	polycarbonate
CNT	carbon nanotube
SWCNT	single-walled carbon nanotube
MWCNT	multi-walled carbon nanotube
BC	bacterial cellulose
DC	direct current
FMDV	foot-and mouth disease virus
pHEMA	poly(2-hydroxyethyl methacrylate)
[BMIM]Cl	1-butyl-3-methylimidazolium chloride
DNA	deoxyribonucleic acid
PLA	polylactic acid
GDH	glucose dehydrogenase
GDI	glutar dialdehyde
HRP	horseradish peroxidase
CNF	cellulose nanofibrils
PM	physical mixing
CI	chemical interaction
GO	graphene oxide
CA	cellulose acetate
IL	ionic liquid
CNC	cellulose nanocrystal
ABTS	2,2’-Azino-bis(3-ethylbenzothiazoline-6-sulfonic acid)
PVA	polyvinyl alcohol
rGO	reduced graphene oxide
CMC	carboxymethyl cellulose
BA	boric acid
BET	Brunauer Emmett Teller
CVD	chemical vapour deposition
GOx	glucose oxidase
PANI	polyaniline
NP	nanoparticle
ExG	exfoliated graphite
